# FOXP1 functions as an oncogene in promoting cancer stem cell-like characteristics in ovarian cancer cells

**DOI:** 10.18632/oncotarget.6510

**Published:** 2015-12-09

**Authors:** Eun Jung Choi, Eun Jin Seo, Dae Kyoung Kim, Su In Lee, Yang Woo Kwon, Il Ho Jang, Ki-Hyung Kim, Dong-Soo Suh, Jae Ho Kim

**Affiliations:** ^1^ Department of Physiology, School of Medicine, Pusan National University, Yangsan 626-870, Gyeongsangnam-do, Republic of Korea; ^2^ Department of Obstetrics and Gynecology, School of Medicine, Pusan National University, Yangsan 626-870, Gyeongsangnam-do, Republic of Korea; ^3^ Research Institute of Convergence Biomedical Science and Technology, Pusan National University Yangsan Hospital, Yangsan 626-770, Gyeongsangnam-do, Republic of Korea

**Keywords:** cancer stem cells, epithelial ovarian cancer, FOXP1

## Abstract

Ovarian cancer has the highest mortality rate of all gynecological cancers with a high recurrence rate. It is important to understand the nature of recurring cancer cells to terminally eliminate ovarian cancer. The winged helix transcription factor Forkhead box P1 (FOXP1) has been reported to function as either oncogene or tumor-suppressor in various cancers. In the current study, we show that FOXP1 promotes cancer stem cell-like characteristics in ovarian cancer cells. Knockdown of FOXP1 expression in A2780 or SKOV3 ovarian cancer cells decreased spheroid formation, expression of stemness-related genes and epithelial to mesenchymal transition-related genes, cell migration, and resistance to Paclitaxel or Cisplatin treatment, whereas overexpression of FOXP1 in A2780 or SKOV3 ovarian cancer cells increased spheroid formation, expression of stemness-related genes and epithelial to mesenchymal transition-related genes, cell migration, and resistance to Paclitaxel or Cisplatin treatment. In addition, overexpression of FOXP1 increased promoter activity of ABCG2, OCT4, NANOG, and SOX2, among which the increases in ABCG2, OCT4, and SOX2 promoter activity were dependent on the presence of FOXP1-binding site. In xenotransplantation of A2780 ovarian cancer cells into nude mice, knockdown of FOXP1 expression significantly decreased tumor size. These results strongly suggest FOXP1 functions as an oncogene by promoting cancer stem cell-like characteristics in ovarian cancer cells. Targeting FOXP1 may provide a novel therapeutic opportunity for developing a relapse-free treatment for ovarian cancer patients.

## INTRODUCTION

Among all gynecologic cancers, ovarian cancer is a highly fatal disease and a major contributor to cancer-related death in women [1]. Ovarian cancers are asymptomatic or show vague symptoms in the early stage, thus most ovarian cancers are detected at advanced stages after cancer has spread beyond the primary tumor site [2]. The standard treatment for ovarian cancer is based on the combination of surgery and chemotherapy. However, the relapse rate is high after cytoreductive surgery and systemic chemotherapy. Therefore, understanding the mechanism of metastasis and resistance to chemotherapy in ovarian cancer is very important in order to improve clinical outcomes.

Previous studies reported that ovarian cancer contained a small fraction of cells that exhibited enhanced tumor initiating potential and stem cell-like properties, thus called cancer stem cells (CSCs) [3–5]. CSCs derived from ovarian cancer are characterized by their self-renewing capability, high tumorigenicity, and resistance to chemotherapy and radiotherapy [6]. CSCs have been suggested as the cause of tumor metastasis and tumor relapse because patients with residual ovarian CSCs ultimately acquire chemoresistance after standard combination of surgery and chemotherapy [7, 8]. CSCs have also been reported to exhibit epithelial to mesenchymal transition (EMT), which enhances motility and invasiveness of cancer cells [9]. However, the molecular mechanisms involved in the contribution of CSCs to chemoresistance and relapses in ovarian cancer have not been clearly identified. Therefore, molecular understanding of CSC characteristics in ovarian cancer is of critical importance in development of relapse-free treatment for ovarian cancer patients.

The winged helix transcription factor Forkhead box P1 (FOXP1) is one of four members of the subfamily P in the FOX transcription factor family. FOXP transcription factor is widely expressed and plays a key role in development of the brain, heart, and lung in mammals. Embryonic stem cell-specific isoform of FOXP1 is known to stimulate the expression of transcription factor genes required for maintaining pluripotency, such as OCT4, NANOG, NR5A2, and GDF3 [10]. In cancer cells, FOXP1 has been suggested as both a potential oncogene and tumor suppressor depending on the cellular context. High expression of FOXP1 in B cell lymphoma or hepatocellular carcinoma was shown to play a role as an oncogene, showing correlation with the worse outcome [11, 12]. On the other hand, loss of FOXP1 expression was reported in renal cell carcinoma, prostate cancer, lung cancer, and endometrial cancer, with an association with the worse outcome [13–17]. However, in ovarian cancer, the precise role of FOXP1 in CSCs has not been clearly characterized.

In this study, we explored the role of FOXP1 in ovarian cancer by examining the effects of FOXP1 expression on ovarian cancer cells. FOXP1 expression in ovarian CSCs showed functional correlation with CSC characteristics including spheroid formation, proliferation, migration, EMT, and drug resistance. In addition, overexpression of FOXP1 led to up-regulated expression of ABCG2, OCT4, NANOG, and SOX2 genes. These results strongly suggest that FOXP1 plays a role as an oncogene in ovarian cancer and may be a valuable target for development of therapeutics for treatment of CSCs in ovarian cancer.

## RESULTS

### FOXP1 expression increases during spheroid culture of ovarian cancer cells

**Figure 1:**
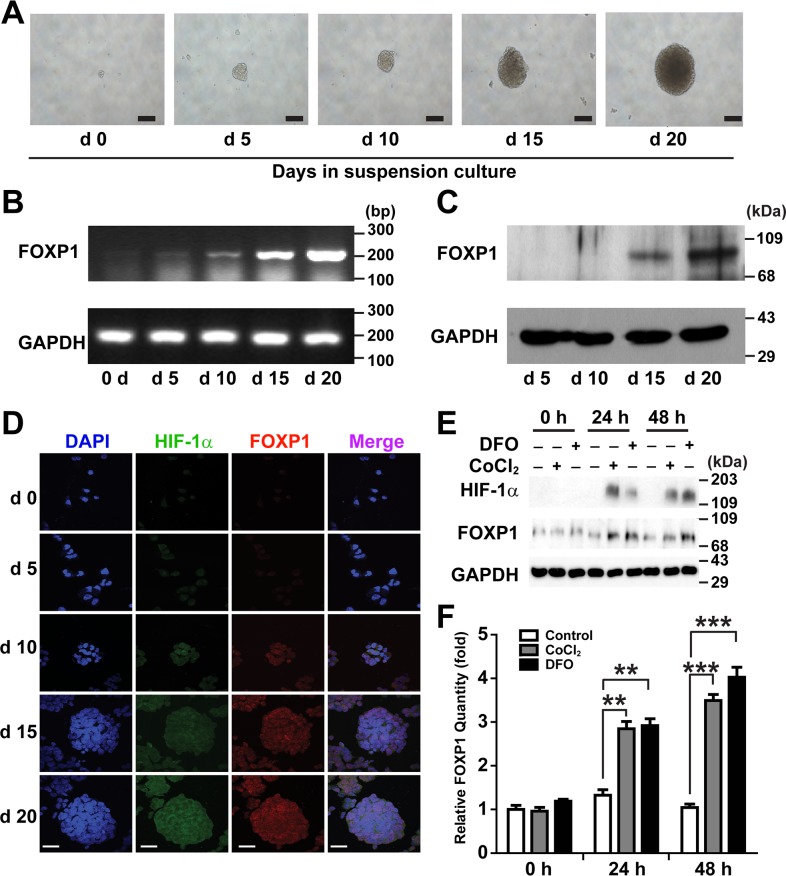
Expression of FOXP1 increases in suspension culture of A2780 ovarian cancer cells **A.** Bright field images of the spheroid generated from A2780 ovarian cancer cells are shown from day 0 to day 20 of spheroid culture (magnification, ×100, bar = 100 μm). **B.** Expression of FOXP1 mRNA in the spheroids generated from A2780 ovarian cancer cells is shown by RT-PCR from day 0 to day 20 of spheroid culture. **C.** Expression of FOXP1 protein in the spheroids generated from A2780 ovarian cancer cells is shown by Western blotting from day 5 to day 20 of spheroid culture. **D.** Expressions of HIF-1α and FOXP1 in the spheroids generated from A2780 ovarian cancer cells are shown by immunocytochemistry from day 0 to day 20 of spheroid culture (bar = 50 μm). **E.** Results of Western blotting analysis after treating A2780 spheroid cells with DFO (100 μM) or cobalt chloride (100 μM) are shown. **F.** Results of quantitative analysis of FOXP1 expression in **E.** are shown.

To determine whether expression of FOXP1 is related to development of CSCs in ovarian cancer, the expression levels of FOXP1 during the spheroid culture of A2780 ovarian cancer cells were evaluated. When A2780 cells were dissociated into single cells and seeded into culture plates with a non-adhesive coating, spheroids, known to contain the enriched CSC population [6], appeared after 5-10 days of culture (Figure 1A). Spheroids showed steady growth over time until day 20. Expression of FOXP1 was analyzed by RT-PCR and Western blotting on day 5, 10, 15, and 20 of spheroid culture. Expression of FOXP1 at mRNA level and protein level started to increase significantly on day 15 and further increased on day 20 (Figure 1B and 1C). When spheroids were subjected to immunocytochemistry analysis, FOXP1 expression increased along with the transcription factor hypoxia-inducible factor-1α (HIF-1α) inside spheroids (Figure 1D). To evaluate if hypoxia contributes to expression of FOXP1, A2780 spheroid cells were treated with Deferoxamine Mesylate/desferal (DFO) or cobalt chloride, which have been known to increase expression of HIF-1α [18]. Treatment of A2780 cells with DFO or cobalt chloride led to increased expression of not only HIF-1α but also FOXP1 (Figure 1E and 1F). These results indicate a correlation of expression level of FOXP1 with development of spheroids and hypoxia in A2780 ovarian cancer cells.

### FOXP1 promotes the spheroid formation of ovarian cancer cells

**Figure 2:**
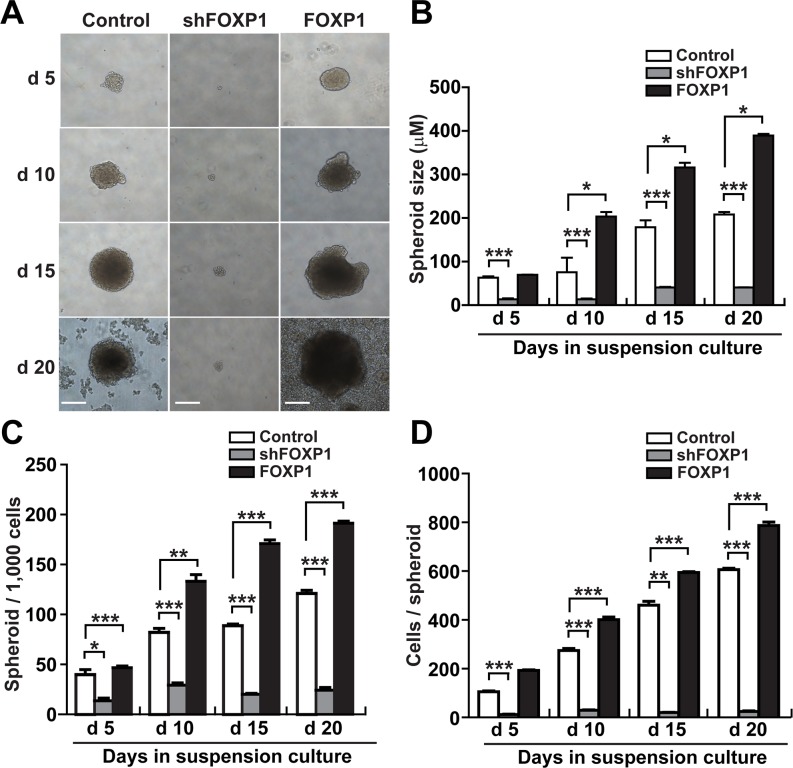
FOXP1 promotes the spheroid formation of A2780 ovarian cancer cells **A.** Bright field images of spheroids generated from A2780 ovarian cancer cells with or without FOXP1 knockdown (shFOXP1) or overexpression (FOXP1) are shown from day 5 to day 20 of spheroid culture (bar = 100 μm). **B.** The size of spheroids generated from A2780 ovarian cancer cells with or without FOXP1 knockdown or overexpression was measured from day 5 to day 20 of spheroid culture. **C.** The number of spheroids generated from 1000 cells of A2780 ovarian cancer cells with or without FOXP1 knockdown or overexpression is shown from day 5 to day 20 of spheroid culture. **D.** The number of cells per spheroid generated from A2780 ovarian cancer cells with or without FOXP1 knockdown or overexpression is shown from day 5 to day 20 of spheroid culture. Data are presented as mean ± SD. *, *p* < 0.05; **, *p* < 0.01; ***, *p* < 0.001.

To determine whether FOXP1 is involved in the regulation of CSC-like characteristics, we tested the effect of FOXP1 knockdown or overexpression on spheroid formation in A2780 and SKOV3 ovarian cancer cells. Knockdown of FOXP1 expression by infecting A2780 cells or SKOV3 cells with lentiviral shRNA against FOXP1 resulted in dramatically reduced size of spheroids in comparison with control spheroids (Figure 2A and 2B and Supplementary Figure 1). By contrast, A2780 cells or SKOV3 cells infected with viruses containing overexpression construct of FOXP1 generated noticeably larger spheroids (Figure 2A and 2B and Supplementary Figure 1). When the numbers of spheroids generated from A2780 cells were counted, A2780 cells with FOXP1 knockdown showed a significant decrease in spheroid numbers, whereas A2780 cells with FOXP1 overexpression showed a significant increase in spheroid numbers compared with those from control A2780 cells (Figure 2C). When the number of cells per spheroid was counted, spheroids from FOXP1-silenced A2780 cells showed dramatically fewer cells, whereas spheroids from FOXP1-overexpressing A2780 cells showed a significant increase in cell number (Figure 2D). These results indicate that FOXP1 plays a regulatory role in formation of spheroids from A2780 ovarian cancer cells.

### FOXP1 promotes expression of stemness-related and EMT-related genes in ovarian cancer cells

**Figure 3:**
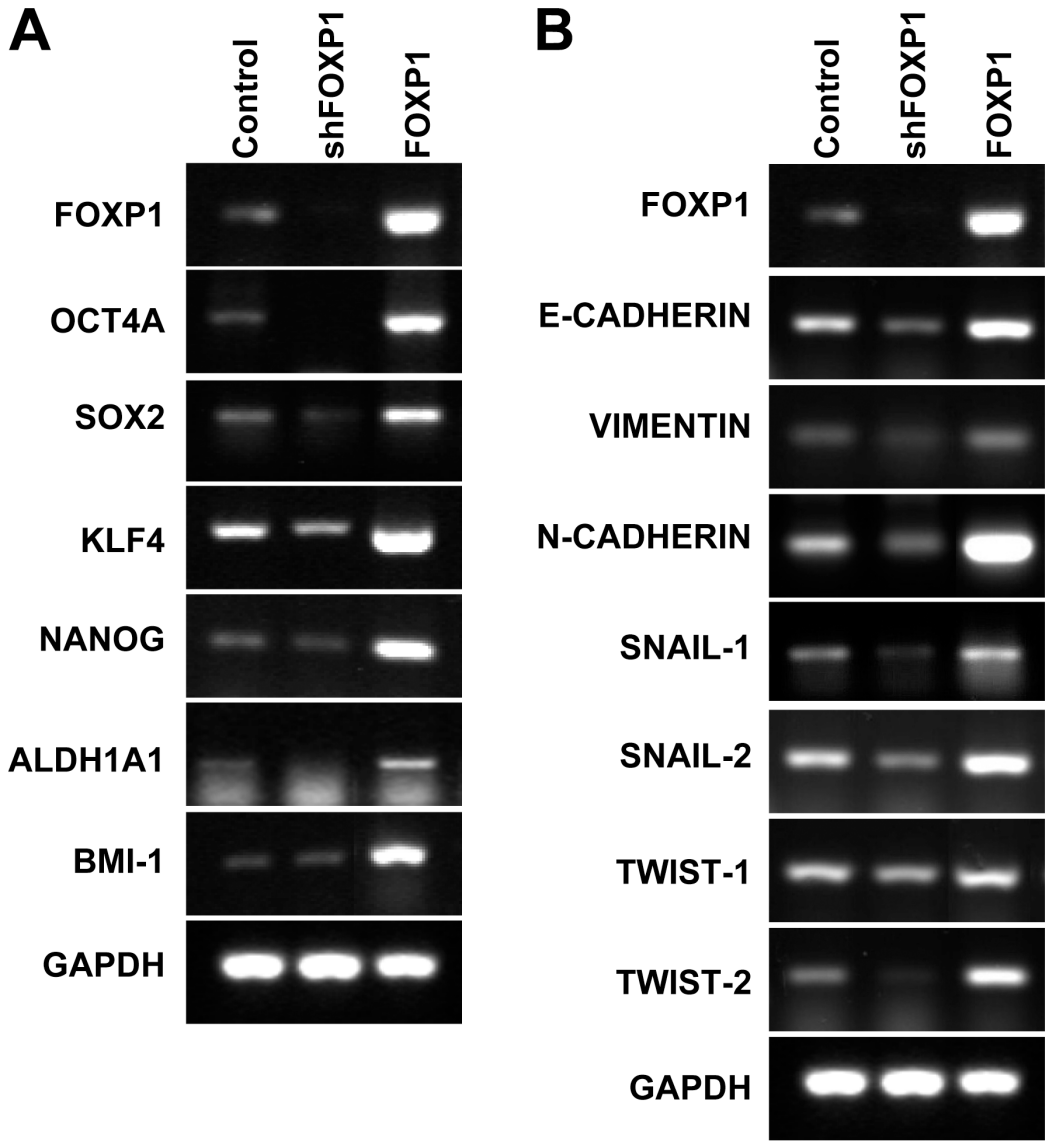
FOXP1 promotes expression of stemness-related genes and EMT-related genes RT-PCR analysis of A2780 ovarian cancer cells with or without FOXP1 knockdown (shFOXP1) or overexpression (FOXP1) was performed using probes for stemness-related genes **A.** or EMT-related genes **B.**

Expression of stemness- or CSC-related genes was analyzed by RT-PCR in A2780 cells and SKOV3 cells after FOXP1 knockdown or FOXP1 overexpression. As shown in Figure 3A and Supplementary Figure 2A, FOXP1 knockdown decreased expression of stemness-related genes including OCT4, SOX2, KLF4, and ADLH1A1 in A2780 cells and SKOV3 cells. On the contrary, overexpression of FOXP1 showed up-regulation of stemness- or CSC-related genes including OCT4, SOX2, KLF4, NANOG, ALDH1A1, and BMI-1 compared with control spheroid cells (Figure 3A and Supplementary Figure 2A). To evaluate if FOXP1 is expressed in ALDH-positive cells, ALDH^high^ and ALDH^low^ cells were isolated from A2780 spheroid cells and subjected to Western blotting analysis. As shown in Supplementary Figure 3, strong expressions of FOXP1 and ALDH1A were detected in non-isolated spheroid cells and ALDH^high^ cells, but not in ALDH^low^ cells. These results suggest that expression of FOXP1 in ovarian cancer cells is required for maintaining and inducing expression of stemness- or CSC-related genes.

To evaluate the effect of FOXP1 expression on EMT of ovarian cancer, expressions of EMT-related genes were analyzed in A2780 cells and SKOV3 cells with knockdown or overexpression of FOXP1. Knockdown of FOXP1 expression significantly decreased expression of E-CADHERIN, VIMENTIN, N-CADHERIN, SNAIL-1, SNAIL-2, TWIST-1, and TWIST-2, whereas overexpression of FOXP1 significantly increased expression of E-CADHERIN, VIMENTIN, N-CADHERIN, SNAIL-1, SNAIL-2, TWIST-1, and TWIST-2 in comparison with control cells (Figure 3B and Supplementary Figure 2B). These results suggest that FOXP1 stimulates expression of EMT-related genes in ovarian cancer cells. Taken together, the results suggest that FOXP1 expression is positively correlated with expression of genes related to CSC-like characteristics in in ovarian cancer cells.

### FOXP1 promotes proliferation and migration of ovarian cancer cells

**Figure 4:**
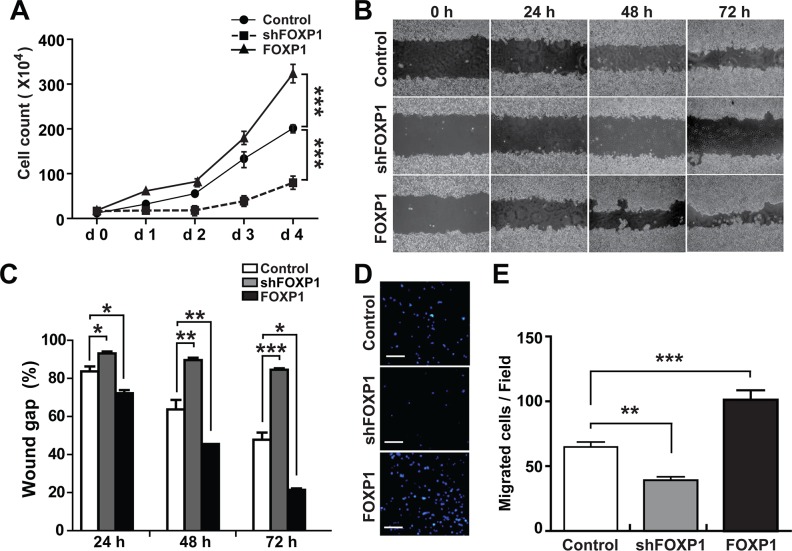
FOXP1 promotes proliferation and migration of A2780 ovarian cancer cells **A.** Cell proliferation was measured by counting cells every day for four days after plating the same number (1×10^4^/well in 12-well culture plate) of A2780 ovarian cancer cells with or without FOXP1 knockdown (shFOXP1) or overexpression (FOXP1). **B, C.** Migration of A2780 ovarian cancer cells with or without FOXP1 knockdown or overexpression was measured by scratch wound healing assay. Bright field images (B) and quantification of wound gap (C) at 24 h, 48 h, and 72 h after application of scratch wound are shown. Wound gap was expressed as a percentage of initial wound gap. **D, E.** Migration of A2780 ovarian cancer cells with or without FOXP1 knockdown or overexpression was measured by transwell migration assay. Fluorescence microscope images of the cell migration (bar = 100 μm) (D) and quantification of migrated cells (E) at 12 h are shown.

To determine whether FOXP1 is involved in the progression of aggressiveness in ovarian cancer, we tested the effect of FOXP1 expression on proliferation and migration of ovarian cancer cells. To evaluate the effect of FOXP1 expression on cell proliferation, A2780 cells or SKOV3 cells with FOXP1 knockdown or FOXP1 overexpression were cultured in comparison with control cells, and cell numbers were monitored for 4 days. As shown in Figure 4A and Supplementary Figure 4A, A2780 and SKOV3 cells infected with lentiviruses against FOXP1 showed a significant decrease of proliferation, whereas FOXP1-overexpressing cells showed an increase in proliferation in comparison with control cells. When cell migration was measured by scratch wound healing assay and transwell migration assay, FOXP1 knockdown significantly decreased cell migration whereas FOXP1 overexpression increased cell migration in A2780 cells and SKOV3 cells (Figure 4B–4E and Supplementary Figure 4B-4E). These results suggest that FOXP1 expression stimulates cell proliferation and migration in ovarian cancer cells.

### FOXP1 promotes resistance to chemotherapy in ovarian cancer cells

**Figure 5:**
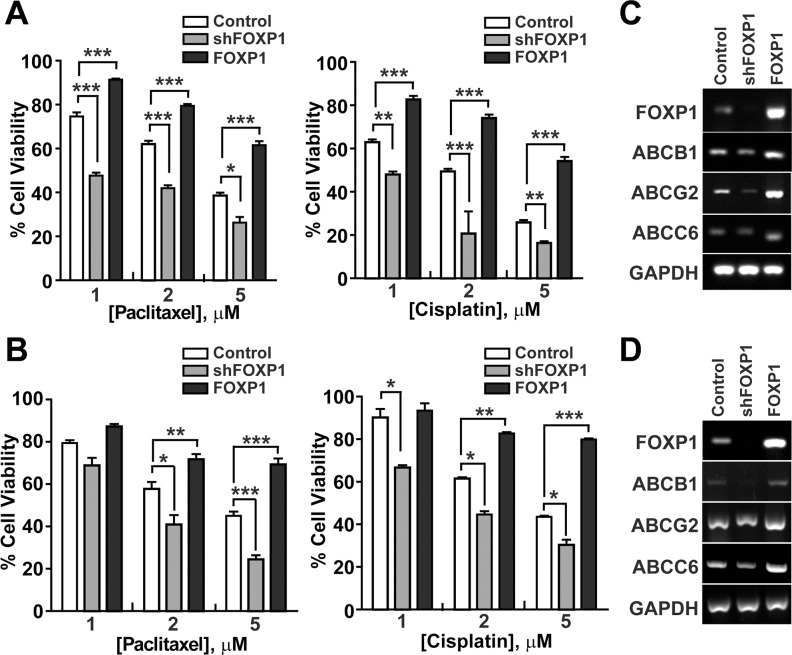
FOXP1 promotes the resistance of ovarian cancer cells to chemotherapeutic drugs **A.** The viability of A2780 ovarian cancer cells with or without FOXP1 knockdown (shFOXP1) or overexpression (FOXP1) was measured by MTT assay after treatment of cells with indicated concentrations of Paclitaxel (left panel) or Cisplatin (right panel). **B.** The viability of SKOV3 ovarian cancer cells with or without FOXP1 knockdown or overexpression was measured by MTT assay after treatment of cells with indicated concentrations of Paclitaxel (left panel) or Cisplatin (right panel). Data are presented as mean ± SD. *, *p* < 0.05; **, *p* < 0.01; ***, *p* < 0.001. **C, D.** Expression of ABC transporters in A2780 (C) or SKOV3 (D) ovarian cancer cells with or without FOXP1 knockdown or overexpression is shown by RT-PCR analysis.

To determine whether FOXP1 expression is involved in regulation of chemoresistance, a key characteristic of CSC, in ovarian cancer, the viability of A2780 or SKOV3 ovarian cancer cells was evaluated with FOXP1 knockdown or overexpression in the presence of chemotherapy reagents Paclitaxel or Cisplatin. As shown in Figure 5A and 5B, the viability of A2780 cells or SKOV3 cells showed a gradual decrease with increasing concentrations of Paclitaxel or Cisplatin. Knockdown of FOXP1 expression in A2780 cells or SKOV3 cells further decreased the viability after Paclitaxel or Cisplatin treatment, indicating a significant increase in the drug sensitivity. On the contrary, overexpression of FOXP1 in A2780 cells or SKOV3 cells increased the resistance to Paclitaxel or Cisplatin treatment. Gene expression analysis by RT-PCR showed that knockdown of FOXP1 in A2780 cells or SKOV3 cells resulted in decreased expression of ABCB1, ABCG2, or ABCC6, whereas overexpression of FOXP1 in A2780 cells or SKOV3 cells led to increased expression of ABCB1, ABCG2, or ABCC6 (Figure 5C and 5D). These results suggest that FOXP1 expression positively correlates with expression of drug transporters and resistance to chemotherapeutic reagents in ovarian cancer cells.

### FOXP1 enhances the promoter activity of ABCG2, OCT4, NANOG, and SOX2

**Figure 6:**
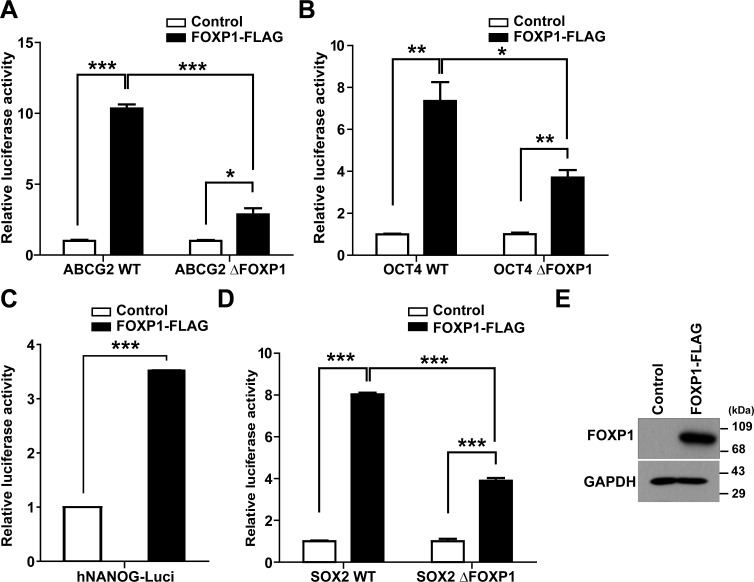
FOXP1 enhances the promoter activity of ABCG2, OCT4, NANOG, and SOX2 **A–D.** Luciferase reporter assay with ABCG2 promoter (A), OCT4 promoter (B), NANOG promoter (C), or SOX2 promoter (D) with or without FOXP1-binding site deletion (ΔFOXP1) was performed after co-transfection of FOXP1 and each reporter construct into A2780 ovarian cancer cells. **E.** Western blotting results of A2780 ovarian cancer cells with or without FOXP1 transfection are shown.

To determine whether FOXP1 directly regulates the expression of genes involved in development of CSC characteristics, the reporter assay was performed with promoters of ABCG2, OCT4, NANOG, or SOX2 after co-transfection of reporter constructs with FOXP1 in A2780 ovarian cancer cells. As shown in Figure 6, overexpression of FOXP1 significantly increased transcription activity of ABCG2, OCT4, NANOG, and SOX2. When FOXP1-binding site was searched in each reporter constructs, promoters of ABCG2, OCT4, and SOX2 showed putative FOXP1-binding sites whereas NONOG promoter did not (Supplementary Figure 5). Deletion of putative FOXP1-bindings sites in ABCG2, OCT4, or SOX2 significantly decreased FOXP1-mediated up-regulation of transcription activity. These results suggest that FOXP1 may up-regulate expression of ABCG2, OCT4, and SOX2 directly and NANOG indirectly in ovarian cancer cells.

### FOXP1 knockdown inhibits the growth of A2780 ovarian cancer cells in xenotransplantation

**Figure 7:**
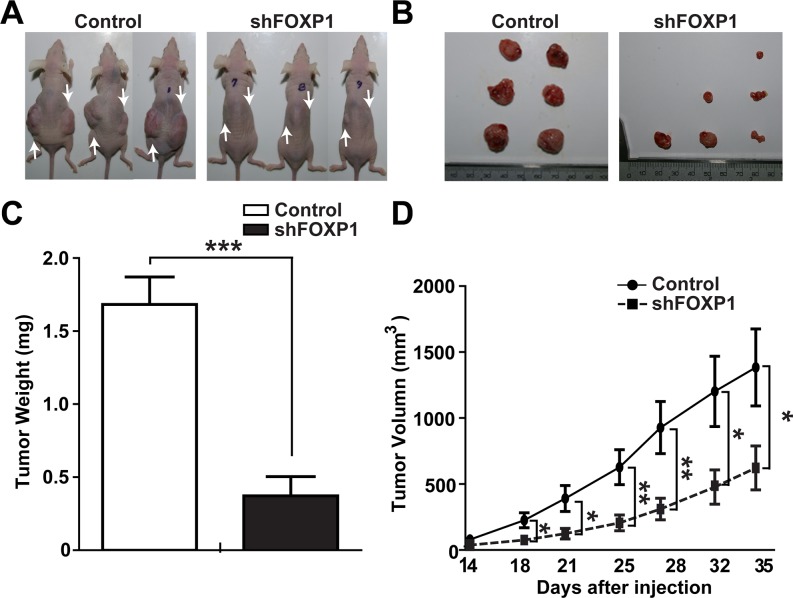
FOXP1 knockdown inhibits tumor growth in xenotransplantation of A2780 ovarian cancer cells **A.** Representative pictures of mice on day 35 after injection of A2780 ovarian cancer cells with or without FOXP1 knockdown are shown. Arrows indicate the injection sites. **B, C.** Representative pictures (B) and the average weight (C) of tumors removed from mice on day 35 after injection of A2780 ovarian cancer cells with or without FOXP1 knockdown into nude mice are shown. **D.** Measurements of tumor volume from day 14 to day 35 after injection of A2780 ovarian cancer cells with or without FOXP1 knockdown into nude mice are shown. Data are presented as mean ± SD (*n* = 6).

To examine the role of FOXP1 in the growth of ovarian cancer *in vivo*, the effect of FOXP1 knockdown on growth of A2780 cells after injection into nude mice was investigated. The same number of A2780 cells with or without FOXP1 knockdown were injected into each side of nude mice, and tumor growth in mice was observed for 35 days. Measureable tumors were detected on day 14 after injection, and tumors were removed on day 35. As shown in Figure 7A and 7B, the injection of control A2780 cells resulted in generation of noticeable sized tumors at the site of injection, whereas the injection of A2780 cells with FOXP1 knockdown generated much smaller tumors. The time course follow-up of tumor volume showed that tumors from A2780 cells with FOXP1 knockdown were consistently smaller than tumors from control A2780 cells during development (Figure 7C). When tumors were weighed on day 35 after sacrificing mice, tumors from A2780 cells with FOXP1 knockdown weighed significantly less than tumors from control A2780 cells. These results suggest that FOXP1 expression is required for *in vivo* growth of ovarian cancer.

Taken together, we showed correlation of FOXP1 expression with the promotion of CSC characteristics, such as the enhancement of spheroid formation, proliferation, and migration, in ovarian cancer cells. FOXP1 expression promoted the expression of genes related to development of CSC characteristics including stemness-related genes, EMT-related genes, and drug resistance-related genes. In addition, FOXP1 knockdown significantly inhibited *in vivo* growth of ovarian cancer cells. These results strongly suggest that FOXP1 functions as an oncogene in ovarian cancer and can be a valuable target in development of relapse-free treatment for ovarian cancer patients.

## DISCUSSION

FOXP1 is known to function as a tumor suppressor or an oncogene in different human cancer types depending on the cellular context. High expression of FOXP1 was reported in a variety of B-cell lymphomas, in which FOXP1 plays an oncogenic role [19]. In contrast, FOXP1 was reported to act as a potential tumor suppressor in prostate cancer, renal cell carcinoma, or breast cancer. [20]. However, the function of FOXP1 in ovarian cancer has not been clear. Previous studies of FOXP1 in ovarian cancer have reported correlation of expression with the degree or malignancies of ovarian cancer along with contradictory observations [15, 21–25]. Recently, in analysis of tissue samples from ovarian cancer patients, Lin's group reported negative correlation of nuclear expression of FOXP1 with increasing tumor grade and poor prognosis, suggesting that FOXP1 may function as a tumor suppressor [23–25]. However, Giatromanolaki et al. found no correlation between FOXP1 and overall survival in low-risk, early-stage endometrial cancers [15]. On the contrary, the prognosis of patients with chemoresistance was very poor when FOXP1 was up-regulated in stage III serous ovarian carcinoma, suggesting that FOXP1 may function as an oncogene [22].

In the current study, we demonstrated that FOXP1 functions as an oncogene in epithelial ovarian cancer cells by promoting the CSC-like characteristics including spheroid formation, cell proliferation, cell migration, drug resistance, and tumorigenic potential. In estrogen receptor α-positive MCF-7 breast cancer cells, overexpression of FOXP1 by exogenous transfection increased cell proliferation whereas knockdown of FOXP1 expression by siRNA decreased proliferation of MCF-7 cells [26]. In glioblastoma with epidermal growth factor receptor amplification, silencing FOXP1 expression inhibited epidermal growth factor receptor-dependent tumorigenicity [27]. In bone marrow-derived Ba/F3 cells, FOXP1 was suggested as a therapy resistance marker as FOXP1 protected cells against apoptotic cell death and knockdown of FOXP1 decreased expression of genes involved in cell migration [28]. These results suggest that FOXP1 functions as an oncogene depending on the cellular context.

Accumulating evidence suggests that epithelial ovarian cancer is a CSC-driven disease [29, 30]. In the current study, we showed FOXP1 up-regulated transcription activity of ABCG2, OCT4, NANOG, and SOX2 in A2780 ovarian cancer cells. The first report of CSCs in epithelial ovarian cancer showed expression of OCT4 and NANOG in self-renewing spheroids [6]. In embryonic stem cells, an evolutionarily conserved alternative splicing variant of FOXP1 was shown to induce the expression of pluripotency genes, including OCT4 and NANOG, and promote the maintenance of pluripotency [10]. However, embryonic stem cell-specific splicing variant of FOXP1 was not detected in A2780 ovarian cancer cells (data not shown). A recent report showed that SOX2 expression was associated with stem cell state in human ovarian carcinoma [31]. Expression of ABCG2, a drug resistance gene, in ALDH-positive cells has been observed in normal stem cells, cancer stem cells, and drug resistant cancers [32]. Though broader spectrum of CSCs may need to be tested to evaluate how common FOXP1 to core CSC transcription network, FOXP1 up-regulates expression of CSC-related core transcription factors, including ABCG2, OCT4, NANOG, and SOX2, in ovarian cancer cells.

Taken together, the current study demonstrated that FOXP1 functions as an oncogene by promoting CSC-like characteristics in ovarian cancer cells. These results suggest that FOXP1 can be a valuable target for development of therapeutics to eliminate CSCs in ovarian cancer.

## MATERIALS AND METHODS

### Materials

RPMI1640 medium was purchased from Welgene (Gyeongsan, South Korea). HBSS (Hank's Balanced Salt Solution), trypsin, fetal bovine serum, and Lipofectamine/Lipofectamine Plus reagent were purchased from Life Technologies, Inc. Culture plates were purchased from Nunc (Roskilde, Denmark). Human ovarian cancer cell line A2780, SKOV3 were purchased from the American Type Culture Collection (Manassas, VA). Cobalt chloride, Deferoxamine Mesylate/desferal (DFO), dimethyl sulfoxide (DMSO), laminin, bisBenzimide H33258 (#14530) were obtained from Sigma–Aldrich (Louis, MO). Antibodies against FOXP1 were purchased from Cell Signaling Technology (#2005S; Beverly, MA). Antibodies against glyceraldehyde-3-phosphate Dehydrogenase (GAPDH) were purchased from EMD Millipore (Billerica, MA). Antibodies against HIF-1α (#610958), ALDH1 (#611194) were purchased from BD Biosciences (San Jose, CA). Secondary antibodies conjugated to HRP were purchased from Santa Cruz Biotechnology.

### Cell culture and spheroid culture

Epithelial ovarian cancer A2780 cells and SKOV3 cells were maintained in RPMI 1640 medium supplemented with 10% fetal bovine serum, 100 U/mL penicillin, and 100 μg/mL streptomycin. Adherent cells were maintained at 37°C in 5% CO_2_ and detached using trypsin/EDTA solution. Spheroids were generated from A2780 cells after plating at a density of 2×10^3^ cells/10 cm^2^ into Ultra-Low Attachment 6-well culture plates (Corning, NY). Spontaneously-generated spheroids were cultured in a serum-free Neuro Basal Medium supplemented with B-27 Supplement, 10 ng/mL basic fibroblast growth factor, 20 ng/mL epidermal growth factor, 2.5 μg/mL amphotericin B, 100 U/mL penicillin, and 100 μg/mL streptomycin. Fresh medium was added every two or three days, and spheroids were cultured for 20 day

### Hypoxic treatment

To prepare DFO and CoCl_2_ stock solutions in RPMI 1640 tissue culture medium (2% FBS), chemicals were dissolved in culture medium (100 mM), followed by filtering (0.22 μm). The resulting solutions were kept at −20°C. Spheroid cells were plated into laminin-coated 6-well tissue plate (1×10^5^/well), and CoCl_2_ and DFO stock solutions were added to cell cultures to be 100 μM in final concentration. After incubation at 37°C for indicated time periods, cell culture medium was removed, and cells were harvested after washing with HBSS solution.

### Western blotting

Cells were lysed in lysis buffer (20 mM Tris-HCL, 1 mM EGTA, 1 mM EDTA, 10 mM NaCl, 0.1 mM phenylmethylsulfonyl fluoride, 1 mM Na_3_VO_4_, 30 mM sodium pyrophosphate, 25 mM β-glycerol phosphate, 1% Triton X-100, pH 7.4). Cell lysates were centrifuged at 1500 rpm for 15 min at 4°C, and supernatants were used for Western blotting. Lysates were resolved by sodium dodecyl sulfate-polyacrylamide gel electrophoresis (SDS-PAGE), transferred onto a nitrocellulose membrane, and then stained with 0.1% Ponceau S solution (Sigma-Aldrich). After blocking with 5% nonfat milk, the membranes were immunoblotted with various antibodies overnight, and the bound antibodies were visualized with horseradish peroxidase-conjugated secondary antibodies, using the enhanced chemiluminescence Western blotting system (ECL, Amersham Biosciences).

### RNA extraction and RT- PCR

Total RNA was extracted from 80–90% confluent cultures using TRIzol reagent (Sigma) and reverse transcribed into cDNA using the Reverse Transcription cDNA Kit (#RT50KN; NanoHelix Co., Ltd). cDNA in 1 μL of the reaction mixture was amplified using the Ready-2×-Go pre-mix PCR kit (#PMD008L; NanoHelix) and 10 pmol each of sense and antisense primers. The thermal cycle profile was as follows: denaturation at 95°C for 30 s, annealing at 54°C for 30 s depending on the primers used, and extension at 72°C for 30 s. Each PCR reaction was carried out for 25-30 cycles. Primer sequences are shown in Supplementary Table 1. PCR products were analyzed by 1% agarose gel electrophoresis.

### Lentiviral and retroviral infection

Lentiviral shRNA construct targeting FOXP1 was purchased from Sigma–Aldrich (Louis, MO). For generation of lentiviral particles, 293FT cells were transfected with the 6.67 μg targeted viral plasmid and lentiviral packaging mix; 5 μg of VSV-G and 3.33 μg of Δ8.9 using 15 μL Lipofectamine/Lipofectamine Plus reagent. For retroviral overexpression of FOXP1, FOXP1 cDNA was subcloned into BamHI and XhoI sites of the pMXs IRES GFP retroviral vector (#RTV-013; Cell Biolabs, Inc.) and then transfected with the 5 μg targeted viral plasmid, 5 μg of VSV and 5 μg of gag/pol into 293FT cells by using Lipofectamine/Lipofectamine Plus reagent. The culture supernatants containing lentivirus or retrovirus were harvested and concentrated using the Lenti-X Concentrator (#631231; Clontech) and Retro-X Concentrator (#631445; Clontech) at 48 h after transfection. For lentiviral or retroviral transduction, A2780 cells were infected with shRNA-bearing lentivirus or pMXs-RNA-bearing retrovirus in the presence of 5 μg/mL polybrene (Sigma-Aldrich). FOXP1-silenced or –overexpressed cells were selected for 10 days with 1μg/mL puromycin and then maintained in RPMI1640 supplemented with 10% FBS and 1 μg/mL puromycin.

### Cell proliferation assay

A2780 cells and SKOV3 cells were seeded in 12-well culture plates at a density of 1×10^4^ cells/well, cultured for 4 days in normal growth medium (RPMI 1640 medium supplemented with 10% fetal bovine serum, 100 U/mL penicillin, and 100 μg/mL streptomycin). Cells were harvested using trypsin/EDTA solution and washed twice with HBSS. Cell numbers were determined by counting viable cells under a microscope after trypan blue staining.

### Chemotherapy resistance assay

To assess chemosensitivity of ovarian cancer cells to Paclitaxel and Cisplatin, cells were seeded at 4×10^3^ cells per well (96-well plates, Corning) in 100 μL RPMI1640 medium supplemented with 2% FBS. Cells were treated with 0 to 5 μM Paclitaxel and Cisplatin for 24 h (*n* = 3 per drug dose). Relative cell numbers were determined by MTT (3-(4, 5-dimethylthiazol-2-yl) 2, 5-diphenyl tetrazolium bromide) assay. Percentage cell survival was expressed relative to vehicle (DMSO)-treated control. After 24 h of drug treatment, cells were stained with 100 μL sterile MTT dye (0.5 mg/mL, Sigma) for 2 h at 37°C, followed by removal of the culture medium and addition of 100 μL DMSO. Absorbance was measured at 570 nm, with 655 nm as the reference wavelength using a micro-plate spectrophotometer (Tecan, Morrisville, NC). The relative percentage of cell viability was calculated by dividing the absorbance of treated cells by that of the control in each experiment.

### Computational searches for FOXP1 sequences

The FOXP1 binding motif was searched in binding sites using the MatInspector application [33], a part of GenomatixSuite software (Genomatix Software GmbH, Germany). The matrices FOXP1_ES.01 (Genomatix Matrix Library 8.2) were used with a core similarity threshold of 0.75 and a matrix similarity threshold of Optimal −0.02. Sequences bearing a match of any of the four matrices were termed FOXP1 sequences. The remaining sequences were classified as FOXP1 and scanned for other TF binding sites motifs contained in the Genomatix Matrix Library using the standard parameters, as described previously [34].

### ABCG2, OCT4, SOX2 promoter and deletion of FOXP1 binding element promoter constructs

The human OCT4 and SOX2 promoter containing the FOXP1 binding elements luciferase report plasmids were cloned by PCR of human genomic DNA (forward: 5′-G CTCAGTCTTTGAGGGGATTGC-3′; reverse: 5′-CGAGAAGGCAAAATCTGAAGC-3′ for OCT4; forward: 5′-GGAAGGAAACTTAGACGAGGC-3′; reverse: 5′-CTTCTCTCCCTTTCTTTCTC-3′ for SOX2) and excising the promoter fragment from the above mentioned products and subcloned into the Kpn1 (NEB #R3142) and HindIII (NEB #R3104) site of the pGL3 basic vector (Promega). ABCG2 promoter construct was a kind donation from Dr Ross [35]. NANOG-Core promoter was purchased from addgene (pNANOG-Luc; Plasmid #25900). Deletion sequences in reporter constructs are as follows; human ABCG2 promoter (TTGATT**TGTT**TTTACTT, TGGAAA**TGTT**TTCATTT); human OCT4 promoter (ATCTAAA**AACA**AGAGGG); human SOX2 promoter (TAGCGAC**AACA**AGAGAA). Deletion mutant constructs were generated using the QuikChange II XL Site-Directed Mutagenesis Kit (Agilent Technologies) according to the manufacture's instruction. The intended mutations were confirmed by sequencing.

### Transactivation activity assay

A2780 (human ovarian cancer cells, ATCC, Rockville, MD, USA) cells were plated in 12-well culture plates at 3×10^5^ cells per well. Transfection experiments were performed 16 hours after cell seeding using Lipofectamine/Lipofectamine plus according to the manufacturer's instructions. Briefly, the transfection mixtures contained 400 ng of reporter construct (ABCG2 reporter constructs, pGL3-basic containing ABCG2 promoter WT and deletion of FOXP1 binding element ABCG2 promoter; OCT4 promoter; deletion of FOXP1 binding element OCT4 promoter; NANOG; SOX2 promoter; deletion of FOXP1 binding element SOX2 promoter) and 400 ng of pCMV-Tag2B FOXP1 expression plasmid, and 10 ng of internal control plasmid (pCMV-RL vector containing Renilla luciferase, Promega). ABCG2 reporter construct was produced as described previously [35]. Twenty-four hours later, cells were harvested, lysed, and centrifuged at 2000 ×g for 3 min at 4°C, and the luciferase activity was determined according to the manufacturer's instructions. All experimental values were averaged from triplicate determinations per experimental condition, and the experiments were performed in triplicate. Subsequently, luciferase activity was measured using the Dual-Luciferase Reporter Assay System (Promega) using VICTOR3 (Perkin Elmer).

### Scratch wound healing assay

Ovarian cancer cells were seeded into 6-well plates and grown to confluence. A scratch wound was introduced on the cell monolayer using a 200 μL pipette tip. The cells were immediately rinsed with HBSS to remove the cell debris and then grown in normal growth media. Images using phase-contrast microscopy were acquired immediately after the scratch at 0 h, 24 h, 48 h, and 72 h for A2780 cells and at 0 h, 3 h, 6 h, 12 h, and 24 h for SKOV3 cells after introducing scratch wound. For quantification, the wound gap was measured using ImageJ software (ver 1.37), and the average wound gaps are shown after normalization to 0h.

### Transwell migration assay

Ovarian cancer cells were harvested with 0.05% trypsin containing 0.02% EDTA, and suspended in a RPMI medium with 10% FBS at a concentration of 1×10^5^ cells/mL. Membrane filters (8-μm pore size) in disposable 96-well chemotaxis chambers (Neuro Probe, Gaithersburg, MD) were pre-coated for 6 h with 20 μg/mL rat-tail collagen at room temperature. Aliquots (50 μL per well) of the cell suspension were loaded into the upper chambers, and RPMI medium with 10% FBS or experimental medium was placed in the lower chamber. After incubation for 12 h at 37°C, the filters were disassembled, and the upper surface of each filter was scraped free of cells by wiping it with a cotton swab. The numbers of cells that had migrated to the lower surfaces of each filter were determined by counting the cells in three different places under the microscope (×100 magnification) after staining with Hoechst 33342 (10 μM).

### Immunofluorescence Staining

For immunofluorescence staining, cells were fixed in 4% paraformaldehyde in PBS for 10 min, washed twice with PBS, and blocked with 1% FBS in PBS for 30 min; all procedures were performed at room temperature. The fixed specimens were incubated with primary antibodies for 1 h, followed by incubation with secondary antibodies for 1 h. Primary antibodies (1:100) were detected by Alexa Fluor 488 and Alexa Fluor 568 conjugated secondary antibodies (1:1000) (Invitrogen, CA). The specimens were finally washed and mounted in Vectashield medium (Vector Laboratories, CA) with 4′,6-diamidino-2-phenylindole for visualization of nuclei. The stained sections were visualized using laser scanning confocal microscopy (Olympus FluoView FV1000).

### Flow cytometry

ALDH activity was determined using the Aldefluor assay kit (STEMCELL Technologies, Vancouver, BC, Canada) as described by the manufacturer. Analysis of fluorescence intensity of the stained cells was performed using a FACSAria-III cell sorter (BD Biosciences, CA). ALDH activity of the sample was determined based on the fluorescence intensity beyond the threshold defined by the reaction with diethylaminobenzaldehyde. To prevent cross-contamination between ALDH^high^ and ALDH^low^ cells, sorting gates of these two populations were set up at least one log apart.

### *In vivo* xenograft transplantation

Animal experiments were performed using a protocol approved by the Pusan National University Institutional Animal Use and Care Committee. BALB/c-nude mice (male, age 8-10 weeks, weight 22-24g) were randomly divided into two groups (six mice in each group). Mice in the control groups received subcutaneous injection with control-shRNA-infected A2780 cells (1×10^3^ cells per 200 mL PBS) and experimental groups received subcutaneous injection of FOXP1-shRNA-infected A2780 cells (1×10^3^ cells per 200 mL PBS). Cells were injected subcutaneously into the right and left side of the flank region of 8-week-old male BALB/c-nude mice. On indicated dates after injection of cells, tumors volumes were determined using an external caliper. The following formula was used for calculation of tumor volume: tumor volume (mm^3^) = tumor length (mm) × tumor width (mm) × tumor width (mm)/2. All mice were sacrificed by cervical dislocation on day 35 after injection. Subcutaneous tumors were surgically excised, weighed, and photographed.

### Statistical analysis

Quantitative data are presented as the mean ± SD (*n* ≥ 3). Statistical significance was calculated using the unpaired Student's *t*-test. *, *p* < 0.05; **, *p* < 0.01; ***, *p* < 0.001 were considered significant.

## SUPPLEMENTARY FIGURES AND TABLES




