# Supplementary material for: FOXP1 functions as an oncogene in promoting cancer stem cell-like characteristics in ovarian cancer cells
Source: Oncotarget. 2015 Dec 9;7(3):3506–19. doi: 10.18632/oncotarget.6510 (PMC4823123; doi:10.18632/oncotarget.6510)
Supplement: Supplementary file 1 [file oncotarget-07-3506-s001.pdf]

SUPPLEMENTARY FIGURES AND TABLES

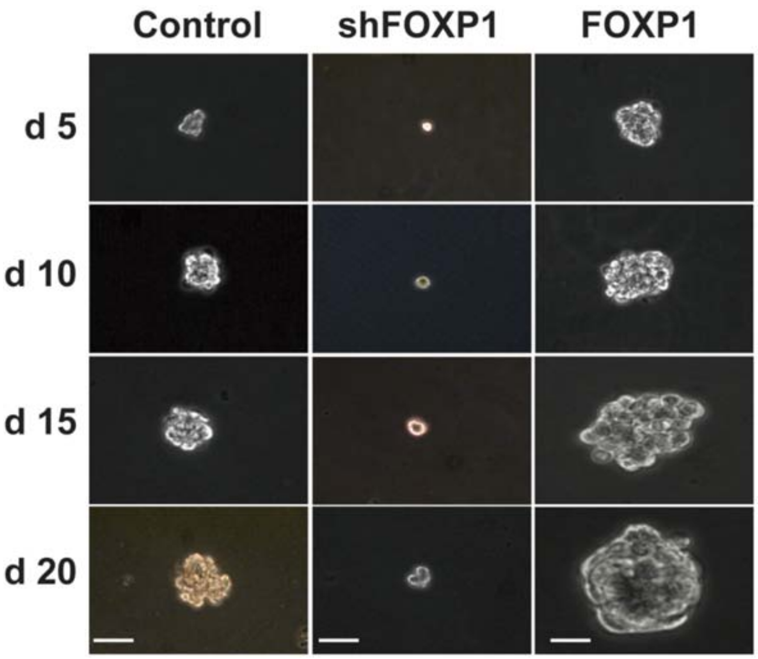

Supplementary Figure S1: FOXP1 promotes spheroid formation of SKOV3 cells. Bright field images of spheroids generated from SKOV3 ovarian cancer cells with or without FOXP1 knockdown (shFOXP1) or overexpression (FOXP1) are shown from day 5 to day 20 of spheroid culture (bar = 100 μm).

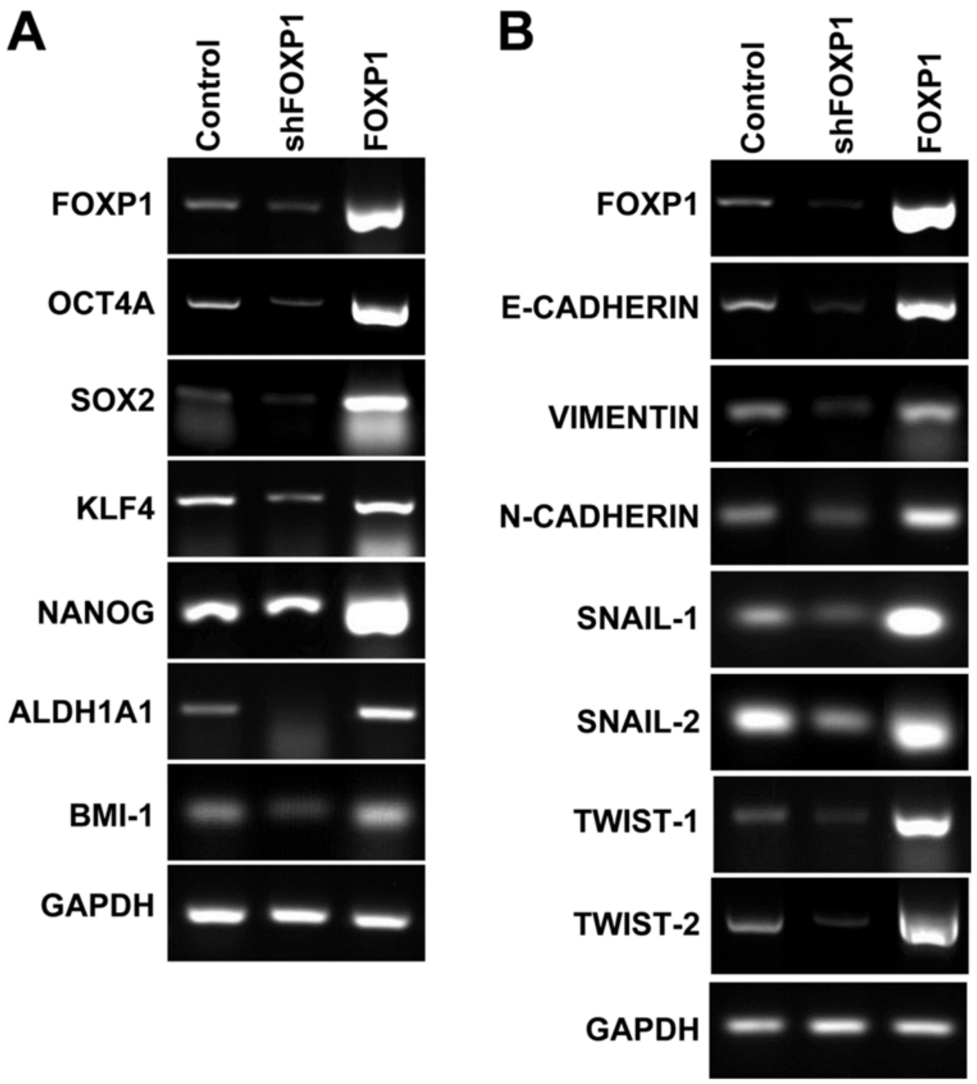

Supplementary Figure S2: FOXP1 promotes expression of stemness-related genes and EMT-related genes in SKOV3 cells. RT-PCR analysis ofSKOV3 ovarian cancer cells with or without FOXP1 knockdown (shFOXP1) or overexpression (FOXP1) was performed using probes for stemness-related genes **A.** or EMT-related genes **B.**.

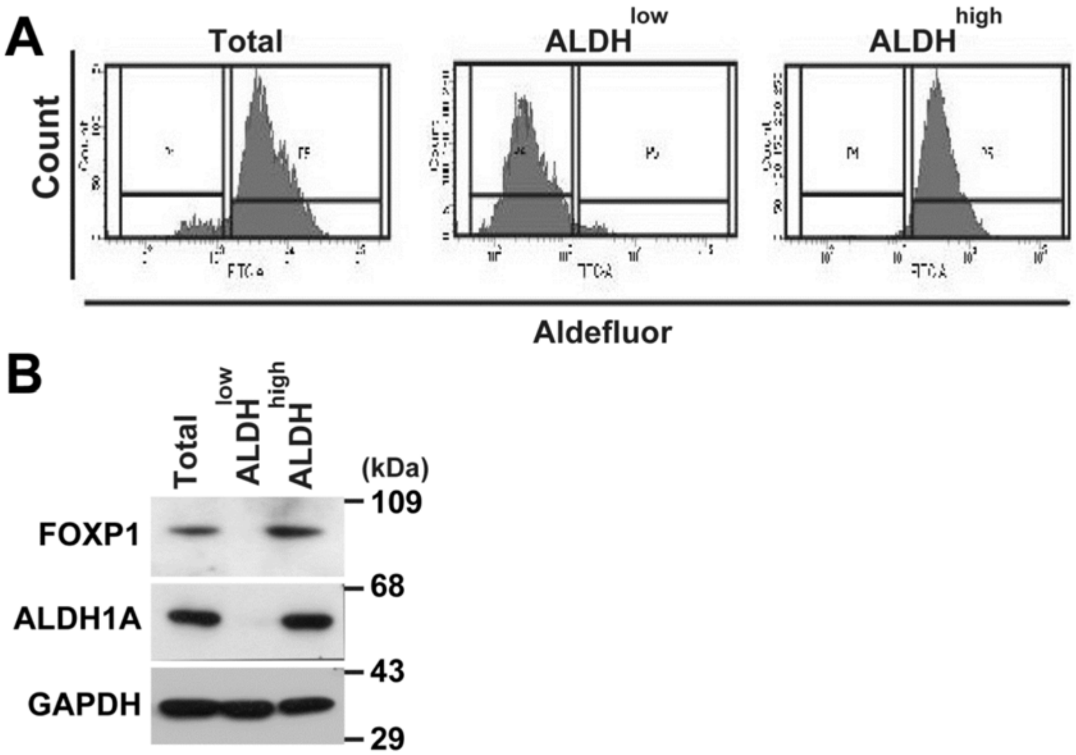

Supplementary Figure S3: FOXP1 is highly expressed in ALDH^{high} cells of A2780 spheroid cells. A. Flow cytometry analysis and sorting of A2780 spheroid cells after incubation with ALDH substrate is shown. **B.** Western blotting analysis of whole spheroid cells before sorting, and ALDH^{low} cells and ALDH^{high} cells after sorting is shown with indicated antibodies.

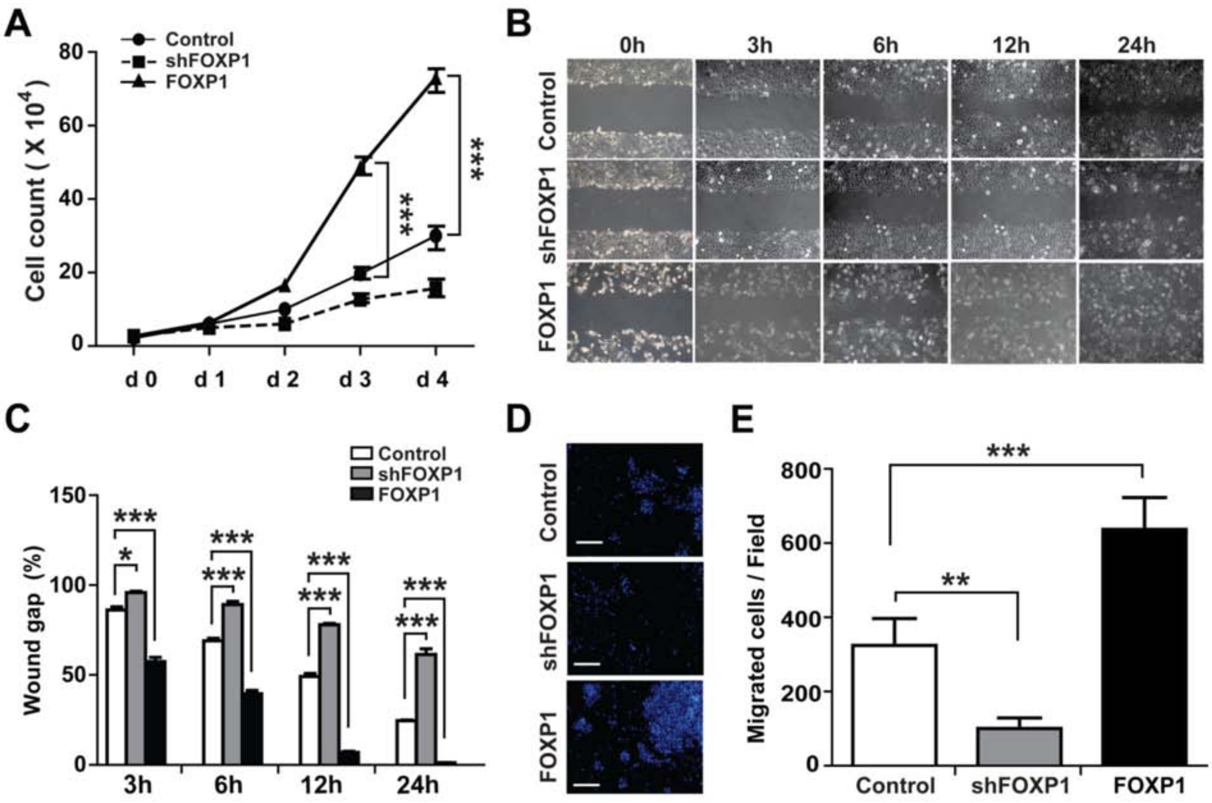

Supplementary Figure S4: FOXP1 promotes cell proliferation and migration ability of SKOV3 cells. A. Cell proliferation was measured by counting cells every day for four days after plating the same number of SKOV3 ovarian cancer cells with or without FOXP1 knockdown (shFOXP1) or overexpression (FOXP1). **B, C.** Migration of SKOV3 ovarian cancer cells with or without FOXP1 knockdown or overexpression was measured by scratch wound assay. Bright field images (B) and quantification of wound gap (C) at 3 h, 6 h, 12 h, and 24 h after application of wound scratch are shown. **D, E.** Migration of SKOV3 ovarian cancer cells with or without FOXP1 knockdown or overexpression was measured by transwell migration assay. Fluorescence microscope images of the cell migration (bar = 100 μm) (D) and quantification (E) at 12 h are shown. Data are presented as mean ± SD. *, $p < 0.05$; **, $p < 0.01$; ***, $p < 0.001$.

ABCG2

-1285 CTTAGCCTCGCGAGTAGCTGGGATTACAGCCTCGCCACCA

-1215 CACCCAGCTAA**TTGATT*TGTT*TTTACTT**CTTTTGTATTTTTAGTAGAGGCAGGGTTTCACCATGTTGGCC

FOXP1 binding element

-1145 AGGCTGGTCTCGAACTCCTGGCCTCAAGTGATCTGCCTGCGTCAGCCTCCC**TGGAAA*TGTT*TTCATTT**AA

FOXP1 binding element

-1075 AGTGCTGGGATTACAGGCATGAGCCACCGTGCTGGCCTTAATTTTGTCTTAAAATGAAGTATGACTAACA

-1005 TGTATTTCTATATGGGCCTTTAAGGGTCTTGAAACTGACAGAAATTAATTCAAAAGAATAATTTTACCCA

OCT4

-260 TGTCTTAAAAAATAAAAATAAAAAAAGTTTCTGTGGGGGACCTGCACTGA

-190 GGTCCTGGAGGGGCGCCAGTTGTGTCTCCCGGTTTTCCCCTTCCACAGACACCATTGCCACCACC**ATCTA**

-120 **AA*AACA*AGAGGG**CGCCTCAGTTTCTCCCCCACCTCCCTCTCCTCCACCCATCCAGGGGGCGGGGCCAGA

FOXP1 binding element

-50 GGTCAAGGCTAGTGGGTGGGACTGGGGAGGGAGAGAGGGGTTGAGTAGTCCCTTCGCAAGCCCTCATTTC

EXON #1

+21 ACCAGGCCCCCGGCTTGGGGCGCCTTCCTTCCCCATGGCGGGACACCTGGCTTCAGATTTTGCCTTCTCG

SOX2

-1510 GGAAGGAAACTTAGACGAGGCTTTGTTTGACTCCGTG**TAGCGACA*ACAA*GAGAA**ACAAAA

FOXP1 binding element

-1440 CTACCTATTTGTAACGGACGTGCTGCCATTGCCCTCCGCATTGAGCGCCTACCTATTGAAATCTTTACGT

-1370 CGGGACAATGGGAGAGCGGCTAAAATTACCCTCTTGGGTCCTGGGCGGGCAAGATTCCTGAGCCCCTACC

Supplementary Figure S5: 5′ flanking region sequences of ABCG2, OCT4 and SOX2 include FOXP1 binding elements are shown.

Supplementary Table S1: List of primer sets used in RT-PCR

See Supplementary File 1
