# Supplementary figures and images for: FOXP1 functions as an oncogene in promoting cancer stem cell-like characteristics in ovarian cancer cells
Source: Oncotarget. 2015 Dec 9;7(3):3506–19. doi: 10.18632/oncotarget.6510 (PMC4823123; doi:10.18632/oncotarget.6510)

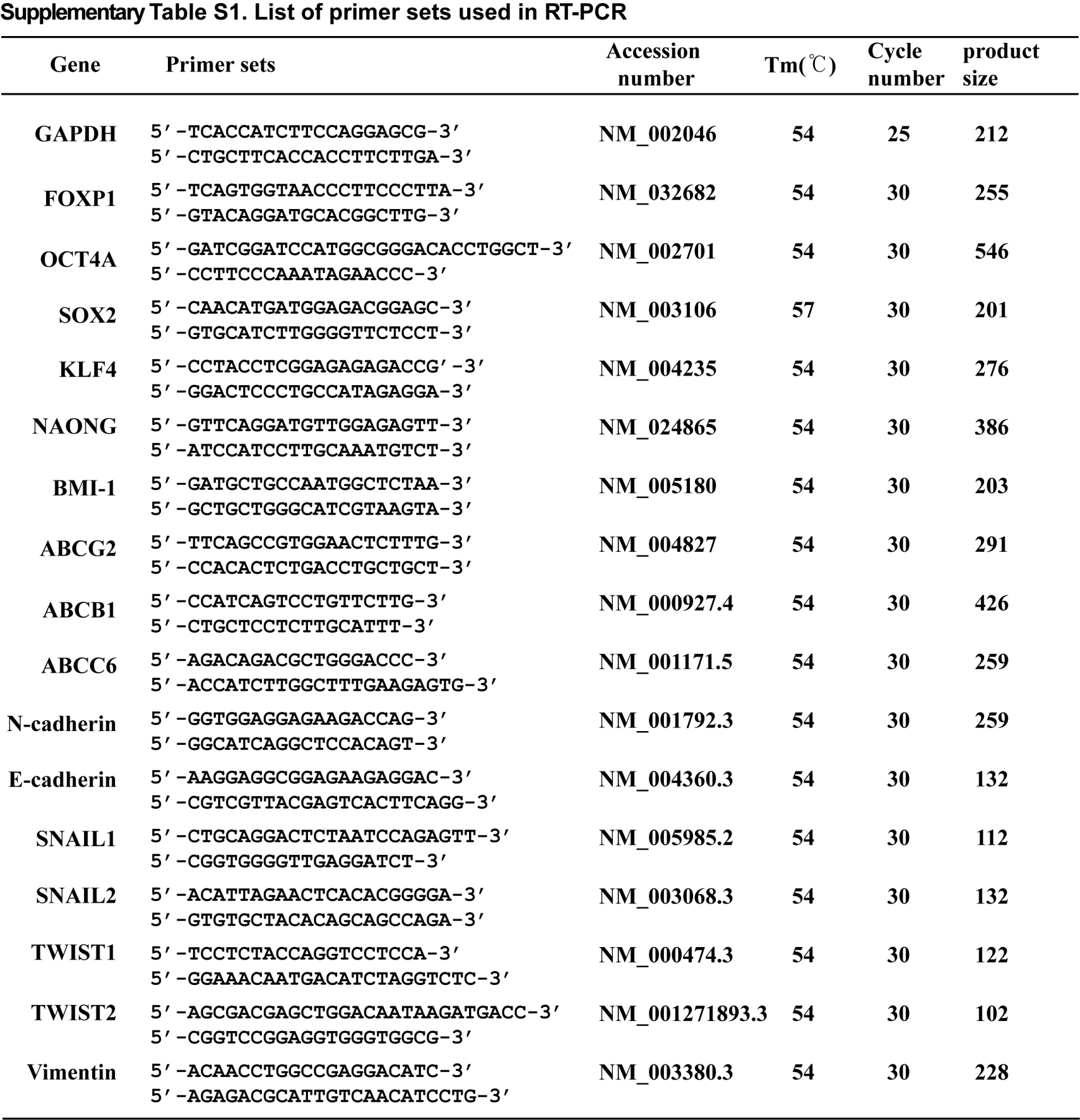

Supplement: Supplementary file 2 [file oncotarget-07-3506-s002.doc]
